# Serum levels of S100B and NSE proteins in Alzheimer's disease patients

**DOI:** 10.1186/1742-2094-7-6

**Published:** 2010-01-27

**Authors:** Márcia L Chaves, Ana L Camozzato, Eduardo D Ferreira, Isabel Piazenski, Renata Kochhann, Oscar Dall'Igna, Guilherme S Mazzini, Diogo O Souza, Luis V Portela

**Affiliations:** 1 Serviço de Neurologia, Hospital de Clínicas de Porto Alegre, Rua Ramiro Barcelos 2350, 90035-003 Porto Alegre, RS, Brazil; 2 Programa de Pós-graduação em Bioquímica, PPG-Bioq. Departamento de Bioquímica, ICBS, UFRGS, Ramiro Barcelos 2600, anexo, 90035-003 Porto Alegre, RS, Brazil

## Abstract

**Background:**

Alzheimer's disease is the most common dementia in the elderly, and the potential of peripheral biochemical markers as complementary tools in the neuropsychiatric evaluation of these patients has claimed further attention.

**Methods:**

We evaluated serum levels of S100B and neuron-specific enolase (NSE) in 54 mild, moderate and severe Alzheimer's disease (AD) patients and in 66 community-dwelling elderly. AD patients met the probable NINCDS-ADRDA criteria. Severity of dementia was ascertained by the Clinical Dementia Rating (CDR) scale, cognitive function by the Mini Mental State Examination (MMSE), and neuroimage findings with magnetic resonance imaging. Serum was obtained from all individuals and frozen at -70°C until analysis.

**Results:**

By comparing both groups, serum S100B levels were lower in AD group, while serum NSE levels were the same both groups. In AD patients, S100B levels were positively correlated with CDR scores (rho = 0.269; p = 0.049) and negatively correlated with MMSE scores (rho = -0.33; *P *= 0.048). NSE levels decreased in AD patients with higher levels of brain atrophy.

**Conclusions:**

The findings suggest that serum levels of S100B may be a marker for brain functional condition and serum NSE levels may be a marker for morphological status in AD.

## Background

Alzheimer's disease (AD) is a progressive brain disorder that results in memory impairment, personality alterations, global cognitive dysfunction, and functional impairments [1]. It is the most common dementia in the elderly, accounting for 60-80% of cases, and it is estimated to affect more than 4 million of USA citizens [2]. The lifespan of individuals diagnosed with AD is reduced by about 50% as compared with those of similar age without disease, and the survival expectancy is negatively associated with the severity of the disease at the time of diagnosis [3]. Furthermore, there is no definitive ante-mortem diagnostic test for AD, and when the clinical diagnosis is made, is difficult to access and following the course of neural cells loss [4].

The inherent hurdles of studying brain tissues in human populations, especially *in vivo*, are the permanent stimulus for finding peripheral markers of central nervous system (CNS) alterations. In this context, a number of proteins have been proposed as peripheral biochemical markers of neuronal damage and glial injury/activation, which peripheral assessment may represent a relevant step forward in the diagnostic and monitoring of CNS diseases [5-9]. For this reason, the clinical usefulness of peripheral biochemical markers as complementary tool in the neuropsychiatric evaluation has claimed further attention.

S100B and neuron specific enolase (NSE) are brain derived proteins extensively studied as peripheral biochemical markers for brain injury [8-11]. S100B is a calcium binding protein physiologically produced and released predominantly by astrocytes, whereas NSE is a cytoplasmatic glycolytic pathway enzyme, being the γγ isoform mainly neuronal [12,13]. Since their levels may increase in CSF and/or blood in several brain pathologies, both proteins are considered to be markers of astrocytic damage/reaction (S100B) and neuronal damage (NSE) [14-16]. Considering the prominent neural death observed in the course of AD, some studies have also attempted to clinically evaluate the levels of these proteins, resulting in contradictory findings [17-21], but alternatively, experimental and human studies have strengthened the belief that S100B is implicated in the mechanisms underlying neurodegeneration in AD [22-24]. Accordingly, it was reported an association between the deposition of cerebral amyloid beta protein and the presence of activated astrocytes over expressing S100B.

Furthermore, life-long over expression of S100B in Down syndrome patients and transgenic mice cause neuronal and glial morphological alterations similar to those found in AD patients, as well as behavioral deficits in animals [17-19]. These conjectures have been the rational for studying CSF/serum S100B and NSE levels in AD as markers of neurodegeneration and severity of the disease. Also, it is important to take into account that, due to the insufficiency of professional, methodological and background conditions, the diagnosis of AD in non specialized centers may not be accurately performed, which encourage identifying potential peripheral biomarkers for AD, aiming an easier, accurate and widespread diagnosis [4].

The major aim of our study was to evaluate serum S100B and NSE levels (more readily assessed than CSF) in AD patients and control elderly individuals without pathological cognitive impairment. In addition, we searched for correlations among their levels and the severity of dementia, cognitive status and brain morphological changes accessed by MRI.

## Methods

### Participants and study design

A cross-sectional study with AD patients and control community-dwelling elderly was carried out. Thirty six AD patients met the probable NINCDS ADRDA criteria [25] and were recruited from the Neurogeriatric outpatient clinic of Hospital de Clínicas de Porto Alegre (HCPA), Porto Alegre, RS, Brazil. Severity of dementia was assigned with the Clinical Dementia Rating (CDR) scale and the cognitive status was assessed by Mini Mental State Examination (MMSE) [26-28]. The CDR is a scale in which CDR = 0 denotes no cognitive impairment, and the remaining points indicate various stages of dementia: CDR = 1 - mild dementia, CDR = 2 - moderate dementia, and CDR = 3 - severe dementia. Exclusion criterion for patients was the presence of any other neurological or psychiatric condition (except if associated with AD), or diseases that could lead to confusion in the diagnosis of AD.

A control group composed of 66 community-dwelling elderly individuals was recruited from the catchment's area of the same hospital. The inclusion criteria were age higher than 60 years and a CDR = 0. Controls were excluded if they presented chronic renal disease, history of significant head injury or stroke; other psychiatric conditions such as major affective disorder or evidence of current depression; uncorrectable vision or hearing loss or other conditions such as substance abuse or use of medications that could impair cognitive function.

AD patients were evaluated with brain magnetic resonance imaging. Patients were additionally submitted to a complete medical and laboratory evaluation. Educational attainment was checked for all participants.

Blood samples (3 ml) for S100B and NSE levels measurement were collected by venipuncture with a tube (vacuum system) without anticoagulants by a trained professional. Serum (was obtained by centrifugation at 5,000 × *g *for 5 min and, soon thereafter, it was frozen at -70°C until analysis.

### Brain MRI

Neuroimaging data were acquired with MRI equipments of 1.5 T (Siemens Magneton Vision Plus or Siemens Symphony, Siemens Medical Systems, Erlang, Germany) with the axial SE and TSE pulse sequences, in T2, (TR: 7,100; TE: 115; slice width: 5 mm; FOV: 230; matrix: 345 × 512), FLAIR (TR: 9,000; TE: 110; slice width: 5 mm; FOV: 230; matrix: 154 × 256) and IR (TR: 1,450, TE: 115, slice width: 3 mm, FOV: 200; matrix: 160 × 256). Sagital SE images were also acquired in T1 (TR: 580; TE: 14; slice width: 5 mm; FOV: 260; matrix: 156 × 256). The average duration of the exam was 30 minutes.

Degree of brain atrophy was measured according to the method of Meese et al. 1980 [29]. Two transversal lines were established in the axial plane of the brain, on TSE pulse sequence, in T2. The first estimated the latero-lateral diameter of the lateral ventricles (D1), and the second, between the parietal bones. The brain atrophy index (BAI) was calculated by the equation BAI = 10 - (D2/D1).

Three degrees of brain atrophy were also established: mild (level 1), moderate (level 2), and severe (level 3), based on the 25, 50 and 75 percentile values of the groups combined.

### Serum S100B and NSE analyses

A quantitative monoclonal two-site immunoluminometric assay LIA-mat Sangtec 100 (BYK-Sangtec, Germany), was used for measuring S100B levels in 100 (L of samples. The immunoluminometric assay is composed of three monoclonal antibodies specific to subunit β of S100 and a tracer antibody, which is bound to isoluminol. Oxidation of isoluminol is started by injection of an alkaline peroxide solution and catalyst solution. The immunological reaction is detected by light reaction [30]. The determinations were carried out in two different experiments. The S100B calibration curve was linear up to 20 (μg/L, and the CVs for duplicates across the entire concentration range for the calibrators and samples were < 5%. The detection limit of the assay is 0.02 (μg/L, as provided by the supplier of the LIA-mat Sangtec100 assay. Internal controls provided by manufacturer was used to determine inter- assay variation, which was also <5%. S100B levels are expressed as mean ± standard deviation.

NSE level was evaluated in serum samples by an electrochemiluminescence assay kit (ECLIA, Roche Diagnostics, USA). This is a quantitative method that uses a monoclonal antibody specific for NSE and labeled with a ruthenium complex, which produces light emission when excited [6]. Reactions and quantification were performed in duplicate by a fully automatized equipment Elecsys-2010 (Roche Diagnostics Corporation^®^). Internal software and controls provided by the manufacturer allow controlling the quality of assay. The NSE calibration curve was linear up to 370 (μg/L), and the CVs for duplicates across the entire concentration range for the calibrators, controls and samples were < 5%. The detection limit was 0.015 μg/L. Serum NSE level is expressed as μg/L (mean ± S.D.).

### Ethical aspects

The study was conducted in accordance with the Declaration of Helsinki, and was approved by the Ethics Committee for Medical Research of the university hospital where it was developed. Informed consent was obtained from the subjects, their nearest relatives, or both.

### Statistical analysis

Descriptive statistics are presented with mean ± standard deviation for parametric variables, and absolute and percentage frequency for categories. Comparison of S100B and NSE serum levels between groups was made using one-way ANOVA with Tukey test, and Student's t test for independent samples. Correlation between S100B and MMSE was performed with Spearman's coefficient. Chi-square test with Yates or Fisher exact correction was used for the analysis of association of CDR and MRI findings categories. Comparison ANOVA followed by Tukey test and Student t test were used to analyze differences between serum levels of S100B (AD and controls) and CDR groups. A *p *value < 0.05 was considered statistically significant. Statistical analyses were carried out with the SPSS 16.0 for Windows.

## Results

### Comparisons between AD and Control group

The demographic, clinical and biochemical characteristics of AD patients and control elderly group are depicted in Table 1. The MMSE and CDR scales were altered in AD patients compared to control group.

**Table 1:** Demographic, clinical and biochemical data of subjects.

	Control group (N = 66)	AD patients (N = 54)	P value
Age (years) (mean ± SD)	76.56 ± 5.46	77.13 ± 7.57	0.773
Gender			
Male (%)	20 (30%)	18 (33%)	0.681
Female (%)	46 (70%)	36 (67%)	
Education (in years)	8.48 ± 5.24	5.23 ± 4.38	0.01
MMSE (mean ± SD)	27.09 ± 2.99	10.98 ± 6.44	0.001
CDR (%)			
0	66 (100%)	-	0.001
1 (mild)	-	12 (22%)	
2 (moderate)	-	22 (41%)	
3 (severe)	-	20 (37%)	
S100B (μg/l) (mean ± SD)	0.21 ± 0.36	0.08 ± 0.06	0.008
NSE (μg/l) (mean ± SD)	9.54 ± 5.28	9.28 ± 3.86	0.832

MMSE: Mini Mental State Examination. CDR: Clinical Dementia Rating

There was a statistically significant difference in serum S100B levels between AD and control group (0.08 ± 0.06 vs. 0.21 ± 0.36, μg/L, respectively; p = 0.008), while the serum NSE levels were similar in both groups (9.28 ± 3.86 vs. 9.54 ± 5.28, μg/L, respectively; p = 0.832). Serum S100B and NSE levels did not vary with age (data not shown).

### Serum S100B and NSE levels in AD patients

**Figure 1:**
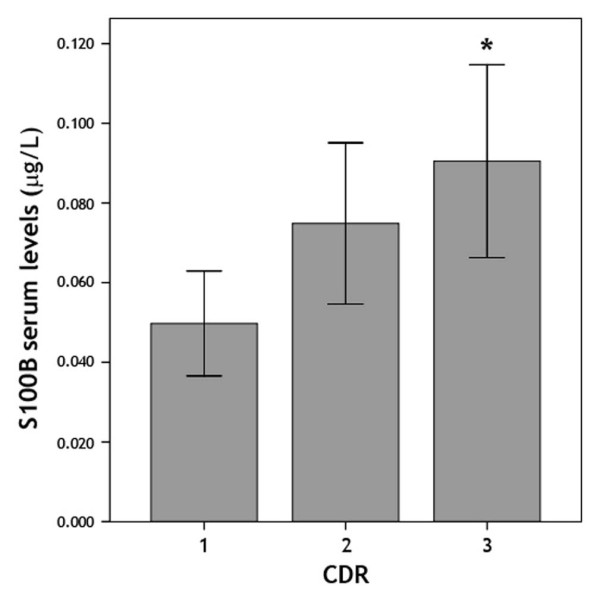
**Serum S100B level (mean ± SD) according to severity of dementia in AD (CDR scale; 1 mild, 2 moderate; 3 severe) - One-way ANOVA (F = 3.685)**.

A positive significant correlation among CDR scores and S100B levels was observed among AD patients (rho = 0.269; p = 0.049, data not shown). Additionally, among AD patients, there was a statistically significant difference in serum S100B levels between mild and severe CDR scores (0.050 ± 0.013 vs. 0.091 ± 0.022, respectively; p = 0.022) (Figure 1). No differences in serum NSE level were observed among CDR categories of AD patients (data not shown).

**Figure 2:**
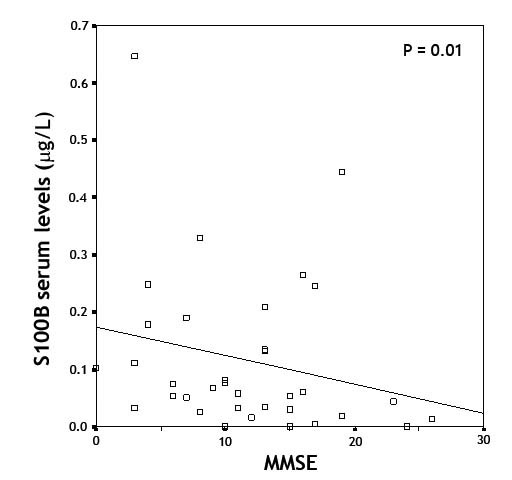
**Correlation between S100B serum levels and Mini Mental State Examination (MMSE) score: AD patients - Spearman rho = - 0.35; P = 0.01**.

There was a significant negative correlation between serum S100B levels and cognitive performance - expressed as a positive correlation between S100B levels and MMSE score (Spearman correlation rho = - 0.35; p = 0.01; Figure 2). A negative correlation between serum NSE levels and MMSE was also observed (Spearman correlation rho = - 0.48; p = 0.017, data not shown).

### MRI and S100 and NSE serum levels in AD patients

Table 2 shows the correlation among serum NSE and S100B levels with the degree of brain atrophy accessed by MRI in AD patients. NSE levels significantly decreased with the brain atrophy severity. Serum S100B levels were not affected by brain atrophy. Despite literature suggest the opposite [31]; there was no correlation among severity of dementia (evaluated by CDR scale) and the MRI findings (Table 3).

**Table 2:** Serum S100B and NSE levels (mean ± SD) according to brain MRI findings in AD patients.

	Degree of brain atrophy		
	Percentil 25 (N = 16)	Percentil 50 (N = 26)	Percentil 75 (N = 12)
**S100B**	0.09 ± 0.05	0.07 ± 0.06	0.07 ± 0.06
**NSE**	14.52 ± 4.08 ^a,b^	7.98 ± 1.52 ^a,c^	6.64 ± 1.17 ^b,c^

a ≠ a: p < 0.001

b ≠ b: p = 0.001

c ≠ c: p = 0.077

**Table 3:** Distribution of dementia severity (CDR categories) according to brain MRI findings in AD patients.

CDR	Degree of brain atrophy		
	Percentil 25 (N = 16)	Percentil 50 (N = 26)	Percentil 75 (N = 12)
**Mild (N = 12)**	3 (19%)	7 (27%)	2 (17%)
**Moderate (N = 22)**	7 (44%)	8 (31%)	7 (58%)
**Severe (N = 20)**	6 (37.5%)	11 (42%)	3 (25%)

Chi-square Fisher Exact = 2.67; p = 0.632

## Discussion

The main goal of this work was to investigate the course of brain neurodegenerative processes in AD patients through biochemical brain markers, neuropsychological and neuroimaging evaluations. Our main findings were: i) although serum NSE levels were not different between AD patients and control elderly individuals, serum of S100B levels were significantly lower in DA patients; ii) there were a positive correlation between S100B levels and AD severity (evaluated by CDR and MMSE) as well as a negative correlation between NSE levels and the severity of morphological brain alterations, evaluated by MRI.

In the last years the possibility of evaluating brain damage/activity through quantification of neuronal and glial derived proteins (such as S100B and NSE) in peripheral samples has gained appropriate attention in clinical and experimental settings [5,14,20,32]. However, S100B and NSE proteins are also expressed in other non neural cell types under physiological and pathological conditions. Thus, the brain specificity of these proteins has been questionable by some works, including from our laboratory [33], when assessing serum samples. Moreover, recent epidemiological and clinico-pathologic data suggest overlaps between AD and cerebrovascular lesions that may magnify the effect of mild AD pathology and promote progression of cognitive decline or even may precede neuronal damage and dementia [34]. So, we cannot rule out that vascular lesions also could account for increase S100B levels.

Despite the controversies on their brain specificity, S100B and NSE have been investigated in different brain diseases as peripheral markers of therapeutic interventions, as well as of neurological and neuropsychological outcome [14,16,22-24]. Additionally, a recent work suggests that blood-brain barrier permeability may also be damaged even at an early stage of AD indicating different blood-brain-CSF compartmental kinetics [35]. Thus, the leakage of proteins from brain to blood could be facilitated.

There is a considerable number of clinical studies regarding CSF levels of S100B in AD, which from the perspective of cerebral protein release provides more sensitivity and specificity than serum samples, however, the availability of CSF samples is somewhat limited for routine clinical use [20,21,36]. These previous studies showed discrepant results with respect to the differences in S100B levels between AD and control groups and also to clinical associations. While some studies have reported increased CSF levels of S100B in AD patients compared to controls [21,36], others did not find any difference [20]. There are also reports suggesting a positive correlation between CSF S100B levels and MMSE, and a negative correlation with severity of dementia by CDR scale [20] while in others CSF S100B levels did not correlate with impaired cognitive status evaluated by MMSE [21,36].

In contrast to these cited works, here we demonstrated AD patients a positive correlation between serum S100B levels and brain function (evaluated by MMSE and CDR). Ethnic differences, education, prior life style and the predominance of female in our study, compared to others, could account for the differences observed among the previous published results. Considering that in our work MMSE and CDR scales identified patients with impaired cerebral functionality affected by AD and that the S100B levels were higher in more severely affected patients, we preliminarily suggest that serum S100B levels could help to distinguish severity or to follow up the progression of dementia in AD disease, even though the diagnosis is based on clinical investigations. This result may reflect the participation of this protein in the pathogenesis of AD. Indeed, glial cells particularly microglia and astrocytes are able to modulate cerebral plasticity and to protect brain from insults [13]; thus, in this context the increase in S100B could indicate astrocytic reaction to neuronal injury (reactive astrogliosis) in AD patients. Once activated, these cells increase the expression of substances that can participate in the excitotoxicity and inflammatory processes that occur during the evolution of AD [37].

Further, dysfunction of glial cells may promote neurodegeneration and, eventually, the retraction of neuronal synapses, which leads to cognitive deficits [38]. However, our major concern is regarding the decreasing levels of S100B in total AD group compared to controls. This finding was confirmed by an additional experiment with a new set of patients. Interestingly, the severity of CDR score was associated with elevations in serum S100B levels, contrasting to our previous findings with schizophrenic patients [39], when the levels were more elevated in the early onset of disease. In this context, increased astrocytic expression of S100B could be involved in the progression of brain neuropathological changes and behavioral deficits observed in patients and animal models [20,13,40-42]. While in this study S100B levels in AD patients did not correlate with brain morphological changes evaluated by MRI, lower serum levels of NSE were related to higher degree of atrophy and brain macroscopic alterations. Furthermore, this loss was not associated with dementia severity in AD patients. The lack of a correlation with atrophy and cognition reported here is somewhat curious as many recent reports of the literature suggest the opposite [31].

Levels of NSE have been shown to be elevated in acute phase of several disorders of the CNS [43-47]. This data could preliminary suggest that AD patients, prior the onset of symptoms, could have altered NSE levels, which is now reflected by brain atrophy and ventricle enlargement. However, the reports of CSF NSE vary from decreased levels [17], no difference [19] to increased levels [48]. Interestingly, Palumbo et al. 2008 [48] showed that CSF NSE level has the same behavior as the other accepted markers of AD, being correlated with Abeta42 and total protein tau (h-tau).

Taking into consideration, the strong association of the prognosis of AD patients with the severity of dementia at the diagnosis [3], and the difficulty of evaluating the degree of neural cells loss in AD [4], further experimental and clinical studies regarding these serum markers in AD disease should be encouraged.

## Conclusions

In conclusion, we showed that in AD patients the serum S100B levels increased with the severity of the disease whereas decreased serum levels of NSE were associated with increased brain morphological damage.

## Competing interests

The authors declare that they have no competing interests.

## Authors' contributions

MLC designed the study, was responsible for the statistical design of the study, supervised the data collection, and wrote the manuscript. ALC was responsible for carrying out the statistical analysis and helped with reviewing the manuscript. EDF supervised the data collection, was responsible for carrying out the statistical analysis and wrote the manuscript. IP, RK, OD and GSM collected the data and assisted with writing the manuscript. DOS and LVP designed the study, made the biochemical measurements and reviewed the manuscript. All authors read and approved the final manuscript.
